# How to handle use of additional medication for Alzheimer’s Disease during randomised clinical trials?

**DOI:** 10.1002/alz.087763

**Published:** 2025-01-09

**Authors:** Claus Dethlefsen, Peter Johannsen

**Affiliations:** ^1^ Novo Nordisk, Novo Alle, Bagsvaerd Denmark; ^2^ Novo Nordisk A/S, Søborg, 2860 Søborg Denmark

## Abstract

**Background:**

Phase 3 randomized clinical trials within Alzheimer’s Disease (AD) typically last over 18 months. Post‐baseline participants can use additional treatment for Alzheimer’s disease, potentially impacting the cognitive ability as evaluated by the primary endpoint. Consequently, this could overestimate or underestimate the treatment effect, depending on the distribution of usage between treatment arms.

**Method:**

The ICH E9 (R1) addendum’s estimand framework provide a means of precisely defining the clinical question of interest, in particular detailing how intercurrent events such as ‘use of additional AD medication’ should be handled and how to interpret the estimated treatment effect. Donohue *et al* (2020) suggested to use a treatment policy strategy for initiation of symptomatic treatments, based on the observation that placebo subjects did not experience an improvement after initiation of symptomatic treatment. The EMA guideline for Alzheimer’s disease (2018), recommends using a hypothetical strategy for initiation of symptomatic treatment.

**Result:**

Use of additional AD medication will qualify as an intercurrent event only as long as the medication is considered to have an effect. Symptomatic AD medication is assumed to have no effect when the patient is no longer exposed. Therefore, we suggest to allow for the intercurrent event ‘use of additional AD medication’ to have both a start and a stop date, and, when applying a hypothetical strategy, only measurements that are considered affected by the intercurrent event will be substituted by outcomes corresponding to the hypothetical scenario were additional AD medication has not been initiated.

**Conclusion:**

Using estimands clarifies the interpretation of treatment effects in clinical trials allowing usage of additional AD medication.

